# Safety and Effectiveness of Cell Therapy in Neurodegenerative Diseases: Take-Home Messages From a Pilot Feasibility Phase I Study of Progressive Supranuclear Palsy

**DOI:** 10.3389/fnins.2021.723227

**Published:** 2021-10-12

**Authors:** Rosaria Giordano, Margherita Canesi, Maurizio Isalberti, Giovanni Marfia, Rolando Campanella, Daniele Vincenti, Viviana Cereda, Alessandra Ranghetti, Chiara Palmisano, Ioannis Ugo Isaias, Riccardo Benti, Giorgio Marotta, Lorenza Lazzari, Tiziana Montemurro, Mariele Viganò, Silvia Budelli, Elisa Montelatici, Cristiana Lavazza, Araceli Rivera-Ordaz, Gianni Pezzoli

**Affiliations:** ^1^Laboratory of Regenerative Medicine – Cell Factory, Center of Transfusion Medicine, Fondazione IRCCS Ca’ Granda Ospedale Maggiore Policlinico, Milan, Italy; ^2^Parkinson Institute, ASST G. Pini-CTO, Milan, Italy; ^3^Neurologic Rehabilitation Unit, Moriggia Pelascini Hospital, Gravedona ed Uniti, Como, Italy; ^4^Neuroradiology Unit, Fondazione IRCCS Ca’ Granda Ospedale Maggiore Policlinico, Milan, Italy; ^5^Department of Neuroradiology, Neurocenter of Southern Switzerland, Lugano, Switzerland; ^6^Neurosurgery Unit, Fondazione IRCCS Ca’ Granda Ospedale Maggiore Policlinico, Milan, Italy; ^7^Onco-Hematology Unit, Bone Marrow Transplantation Center, Fondazione IRCCS Ca’ Granda Ospedale Maggiore Policlinico and University of Milano, Milan, Italy; ^8^Department of Neurology, University Hospital of Würzburg and Julius Maximilian University of Würzburg, Würzburg, Germany; ^9^Nuclear Medicine Unit, Fondazione IRCCS Ca’ Granda Ospedale Maggiore Policlinico, Milan, Italy

**Keywords:** progressive supranuclear palsy (PSP), Parkinson’s disease, cell therapy, mesenchymal stromal cells (MSCs), posture

## Abstract

Mesenchymal stromal cells (MSCs) are multipotent cells with anti-inflammatory properties. Here we tested the safety of MSCs in patients with progressive supranuclear palsy (PSP; ClinicalTrials.gov: NCT01824121; Eudract No. 2011-004051-39). Seven patients were treated. To improve the safety, protocol adjustments were made during the performance of the study. The objectives of our work were: (1) to assess the safety of MSCs and (2) to identify critical issues in cell therapies for neurodegenerative diseases. Autologous MSCs from the bone marrow of PSP patients were administered through the internal carotid arteries. 1-year survival and number of severe adverse events were considered as safety endpoints. Clinical rating scales, neuropsychological assessments, gait and posture analysis, single-photon emission computed tomography, positron emission tomography, and brain magnetic resonance (BMR) were performed at different follow-up times. Peripheral blood levels of inflammatory cytokines were measured before and after cell infusion. Six of the seven treated patients were living 1 year after cell infusion. Asymptomatic spotty lesions were observed at BMR after 24 h in six of the seven treated patients. The last patient in the preliminary cohort (Case 5) exhibited transiently symptomatic BMR ischemic alterations. No severe adverse events were recorded in the last two treated patients. Interleukin-8 serum concentrations decreased in three patients (Case 2, 3, and 4). An adaptive study design, appropriate and up-to-date efficacy measures, adequate sample size estimation, and, possibly, the use of a cellular and/or allogeneic cell sources may help in performing phase II trials in the field.

## Introduction

Progressive supranuclear palsy (PSP) is a sporadic neurodegenerative disease, characterized by ocular motor dysfunction, postural instability, akinesia, cognitive dysfunction, dysarthria, and dysphagia (Steele et al., 1964). The major pathological finding is the accumulation of tau protein in multiple brain regions, including the substantia nigra, globus pallidus, subthalamic nucleus, pontine tegmentum, and cerebellar dentate nucleus (Dickson et al., 2011). Neuroinflammation is an important factor in PSP progression (Bevan-Jones et al., 2019; Inci et al., 2020; Malpetti et al., 2021). Therefore, immunomodulatory disease-modifying therapies may be extremely useful in controlling PSP.

Newly developed, cell-based, advanced therapy medicinal products (ATMPs) have shown encouraging results in different clinical contexts. In particular, mesenchymal stromal cells (MSCs) are currently being used as immunomodulatory drugs in several phase III clinical trials on graft-versus-host disease (GvHD) after bone marrow (BM) transplantation (Gay et al., 2021). MSCs have been granted conditional market approval in Canada and New Zealand for the treatment of children with GvHD refractory to steroids (Cyranoski, 2012).

In 2010, we began preclinical and validation tests to develop treatments for severe and rare neurodegenerative diseases (Fondazione IRCCS Ca’ Granda Ospedale Maggiore Policlinico Ethics Committee approval 1464/10). In 2012, an autologous cell therapy product made of MSCs from the BM of PSP patients was developed following good manufacturing practices (GMP). We performed a phase I/II study [ISS Authorization Prot. N. 37519(11)-PRE 21-1111] to assess the safety and efficacy of MSCs in reducing neuroinflammation and rescuing neuronal survival and function.

In 2016, our group published results from a preliminary phase I study of five PSP patients who were treated with autologous BM MSCs. In the original experimental design, a blind randomized controlled crossover trial would have followed this preliminary phase. In the randomized trial, 20 patients would have been randomly assigned to receive MSC administration followed by a sham intervention or to receive a sham intervention followed by MSC administration, with a 6-month delay between the two interventions. Following the preliminary phase I study, we substantially modified the protocol for cell infusion for safety reasons because intra-arterial MSC administration was associated with an intrinsic risk of microembolization. In our experience, this risk was invariably present in all treated patients, and one patient was symptomatic with transient ischemic brain magnetic resonance (BMR) lesions. To test the effectiveness of these modifications on the treatment safety profile, the Italian National Competent Authority requested that we treat three additional patients in the open, non-randomized phase before moving on to the subsequent phase.

In our study, we administered MSCs by infusion in the carotid arteries to exploit the ability of the cells to pass the blood–brain barrier and to exert their effects directly on brain tissues (Giordano et al., 2014). This form of administration would prevent the MSCs from being entrapped in the lung, as expected if systemic administration was used (Everaert et al., 2012; Sensebe and Fleury-Cappellesso, 2013; De Becker and Riet, 2016). Selective catheterization of the cerebral arteries was previously performed to deliver MSCs and hematopoietic progenitor cells to treat multisystemic atrophy (Lee et al., 2012) and Parkinson’s disease (Brazzini et al., 2010), respectively. The most frequent adverse event recorded in the MSC clinical trial by Lee et al. (2012) was the presence of small ischemic lesions (<1 cm) on diffusion-weighted brain magnetic resonance imaging (MRI). These lesions were associated with the catheterization procedure.

The aims of the present study were to add further data to our previous work (Canesi et al., 2016) in order to assess the safety of intra-arterial administration of autologous MSCs in PSP patients and to identify critical issues and possible solutions for effective cell therapies in treating neurodegenerative diseases. We measured the survival at 1 year from cell administration and the number of severe adverse events as safety endpoints. To examine the effects of the treatment, we used clinical rating scales, neuropsychological assessments, and gait and posture analysis. We measured cytokine profiles in the peripheral blood, but due to logistic and ethical reasons and in consideration of the frailty of the patients, we could not perform liquor collection to assess the effect of MSC administration on cerebral inflammation. In addition, single-photon emission computed tomography (SPECT), positron emission tomography (PET), and BMR were performed before and after treatment. The overall goal of this study was to provide data to guide further phase II studies in the field.

## Method

### Protocol Approval and Patient Screening

This project complied with the mission of our hospital, which is dedicated to regenerative medicine and rare diseases. The protocol was authorized by the local Ethics Committee of Fondazione IRCCS Ca’ Granda Ospedale Maggiore Policlinico (Italy) and the National Competent Authority for phase I cell therapy at the National Health Institute (Istituto Superiore di Sanità). The trial protocol was approved by the Italian Medicines Agency (Agenzia Italiana del Farmaco, AIFA). After the first preliminary phase, subsequent protocol amendments were authorized by the Italian National Competent Authority (AIFA) and by the local Ethics Committees. All patients gave their written informed consent. The trial is registered at ClinicalTrials.gov (NCT01824121). A detailed description of the study design, inclusion and exclusion criteria, BM collection, MSC isolation, and administration, as well as clinical (motor and neuropsychological) and neuroimaging assessments have been previously reported (Giordano et al., 2014; Canesi et al., 2016). The major changes in the protocol conduction were the number of patients to be included (three more in the preliminary phase I study) and the method for cell infusion (see the specific paragraph below).

Initially and periodically (at least once a month), the list of PSP patients was pre-screened by two researchers to select eligible patients based on the information in the most recently updated institutional clinical records. The candidate patients were contacted, and a screening visit was performed to assess the inclusion/exclusion criteria. Information was given to the patient, and informed consent was obtained. The informed consent form was signed by each patient before prior to the commencement of any study-related procedures.

### Cell Manufacturing

Each patient underwent BM harvesting in an outpatient setting. MSC GMP production was performed at the Cell Factory of the public hospital Fondazione IRCCS Ca’ Granda Ospedale Maggiore Policlinico in Milan, Italy. The facility has maintained its manufacturing authorization (AIFA) in compliance with European GMP regulations to produce ATMPs since 2007 without interruption. The facility is a fully controlled plant for ATMP manufacturing; its characteristics as well as the main quality assurance procedures were previously described (Montemurro et al., 2015). Briefly, all manufacturing procedures were performed in a class A environment (Class II Type A2 Biological Safety Cabinet) with a class B surrounding environment. Microbial contamination was monitored using settle plates and volumetric active air sampling, and surfaces and operators were sampled with contact plates. In-continuous airborne particle monitoring was performed in the class A environment and during critical steps in the class B environment using automatic particle counters.

The procedures for manufacturing were developed by the authors. Briefly, unprocessed BM was directly seeded in alpha Modified Eagle Medium supplemented with 10% fetal bovine serum at a concentration of 50,000 total nucleated cells/cm^2^ in a Cell Stack Chamber system (Corning, Lowell, United States). After 72 h, non-adherent cells were removed by washing with phosphate-buffered saline (Macopharma, Mouvaux, France) and completely changing the medium. Medium changes were also performed twice a week. On day 14 (±3), MSCs at P0 were detached using 25 mL/layer of trypsin (TrypLE Select, Gibco-Life Technologies, Carlsbad, CA, United States), transferred in bags, and washed by centrifugation (400 *g*, 12 min, no brake) in normal saline solution with 10% (vol:vol) human serum albumin (Kedrion, Castelvecchio Pascoli, Lucca, Italy). Therefore, the supernatant was discarded and the cells re-seeded in cell stacks in the same culture conditions at a concentration of 4,000 MSCs/cm^2^. The culture was stopped at 28 ± 3 days (passage 2), and the cells were detached and washed as at P0 and re-suspended in a solution containing normal saline solution with 10% (vol:vol) human serum albumin (Kedrion) and 10% (vol:vol) dimethyl sulfoxide (Bioniche Lifesciences, Inc., Belleville, ON, Canada). The cell product was cryopreserved at a concentration of 1–5 × 10^6^ cells/mL using a controlled-rate freezer (Nicool Plus, Air Liquide) programmed to freeze at –1°C/min and stored in bags (CryoMACS Miltenyi, Teterow, Germany) in the vapor phase of liquid nitrogen.

The quality criteria for releasing the final cell product were: number of cells > 1,5 × 10^6^ cells/kg of body weight, purity (% CD90+/105+/45 – cells) > 80%, viability (% P.I. – cells) > 80%; sterility; bacterial endotoxin < 0.25 E.U./mL, absence of Mycoplasma; normal karyotype.

On the day of the infusion, the cells were thawed at 37°C and normal saline solution with 10% (vol:vol) human serum albumin (Kedrion) and 12% anticoagulant citrate dextrose solution, solution A (Fresenius Kabi, Bad Homburg, Germany) were added to the suspension of thawed cells without removing DMSO. The final concentration of DMSO was 1% vol:vol. A visual inspection of the thawed cell suspension was performed using an optic microscope (100×) to exclude cell aggregates, visible impurities, or any other abnormalities just before infusion.

### Cell Infusion

Each patient underwent neuroleptoanalgesia and was monitored by an anesthetist. MSCs were administered by the intra-arterial route, as described previously (Canesi et al., 2016). Briefly, using the Seldinger technique, catheterization was carried out via the right common femoral artery (or the left one in the event of difficulty in achieving arterial access) using a 6F Ultimum EV (St Jude Medical, MN, United States) introducer and a 5F Hinck or Simmons (Terumo Europe NV, Leuven, Belgium) diagnostic catheter.

An angiographic study of the cervical and intracranial arteries was performed using a 0.035-inch diameter, 150-cm long hydrophilic guide (Terumo Europe NV, Leuven, Belgium). Intravenous administration of heparin sodium (3,000–5,000 IU according to body mass) was completed. Subsequently, using a 260 cm exchange wire Easykit with a 0.35″ or 0.38″ diameter [Ab Medica S.P.A., Lainate (MI), Italy], a 90 cm 6F guiding catheter Envoy XB (Miami Lakes, FL, United States) was placed. The guiding catheter, flushed by heparinized saline, was positioned at the origin of both internal carotid arteries and at the origin of the widest vertebral artery. Once the guiding catheter was in place, a microcatheter Rebar 027 (130 or 145 cm) or Rebar 018 (153 cm; ev3/Covidien, Irvine, CA, United States), steered by a 205 cm Transend EX 0.014 (Boston Scientific, Natick, MA, United States), was moved forward up into the internal carotid arteries. The microcatheter was placed just above the origin of the ophthalmic arteries and in the widest vertebral artery up to its V2 segment. The MSCs were then injected into the various locations (the left and right internal carotid arteries and the widest vertebral artery) through the microcatheter using a pump at 70 ± 30 mL/h. The catheter was periodically flushed with heparinized normal saline solution.

### Clinical and Neuropsychological Assessment

Patients were observed for the occurrence of adverse events during each protocol procedure and at all follow-up points.

After arterial catheterization, the patient was strictly monitored with the face arm speech test immediately after the procedure and then 3, 6, 18, and 24 h later with the aim for rapidly recognizing early signs of cerebral ischemia. BMR was performed 24 h after the procedure. If asymptomatic ischemic lesions were observed, anti-platelet therapy was continued and BMR was repeated after 14 days. In the case that no adverse event was reported, the patient was discharged 48 h after cell administration.

The following scales were used to assess treatment efficacy on neurological functions: the Unified Parkinson’s Disease Rating Scale (UPDRS) part III, motor score (Goetz et al., 2008), Hoehn and Yahr (H&Y) staging (Goetz et al., 2004), the PSP rating scale (PSP-RS; Golbe and Ohman-Strickland, 2007), and the Mini-Mental State Evaluation (Folstein et al., 1975). The scales were administered at baseline and at each follow-up point (1, 3, 6, and 12 months after cell administration). The clinical condition was classified as stable when the UPDRS and PSP-RS scores did not diminish by more than 30% compared to baseline and the H&Y staging did not increase by more than one point at the defined follow-up point.

### Neuroimaging and Biomechanical Evaluation

All patients underwent longitudinal neuroimaging assessments, using brain MRI, striatal dopamine transporter SPECT, and PET. SPECT was performed with [I-123] ioflupane, a selective radioligand for dopamine reuptake transporters, and PET was performed with 2-fluoro-2-deossi-D-glucosio, as previously described (Canesi et al., 2016).

The biomechanical evaluation was completed at baseline and at the 6-month follow-up. The kinematic assessment of posture was performed as described in Palmisano et al. (2020). The assessment accounted for the patient’s base of support and anthropometric measurements. For statistical analysis, the individual patients’ measures were located at the tails of the distribution of the group of healthy subjects (below the 10th and above the 90th percentile). Healthy controls were chosen to have the same demographic and anthropometric characteristics of the patients, which have a large impact on kinematic assessment, as previously demonstrated (Palmisano et al., 2020).

### Inflammatory Cytokine Measurements in the Peripheral Blood of Progressive Supranuclear Palsy Patients

Peripheral blood (sera) levels of interleukin (IL)-6, IL-8, tumor necrosis factor-α, IL-1β, IL-5, IL-10, and interferon-γ were measured before and 72 h after MSC administration. Measurements were made using a high-sensitivity planar enzyme-linked immunosorbent assay with a chemiluminescent substrate (CorPlex Human Cytokine Panel 1, Quanterix, MA). All serum samples were collected in the morning (6:00 am to 8:00 am) after overnight fasting. The samples were centrifuged and stored at –80°C within 1 h after blood withdrawal. Data analysis by Wilcoxon matched-pairs signed-rank test was performed using GraphPad Prism version 8.0.0 for Windows (GraphPad Software, San Diego, CA, United States^1^).

## Results

### Patient Selection and Mesenchymal Stromal Cell Characteristics

**FIGURE 1:**
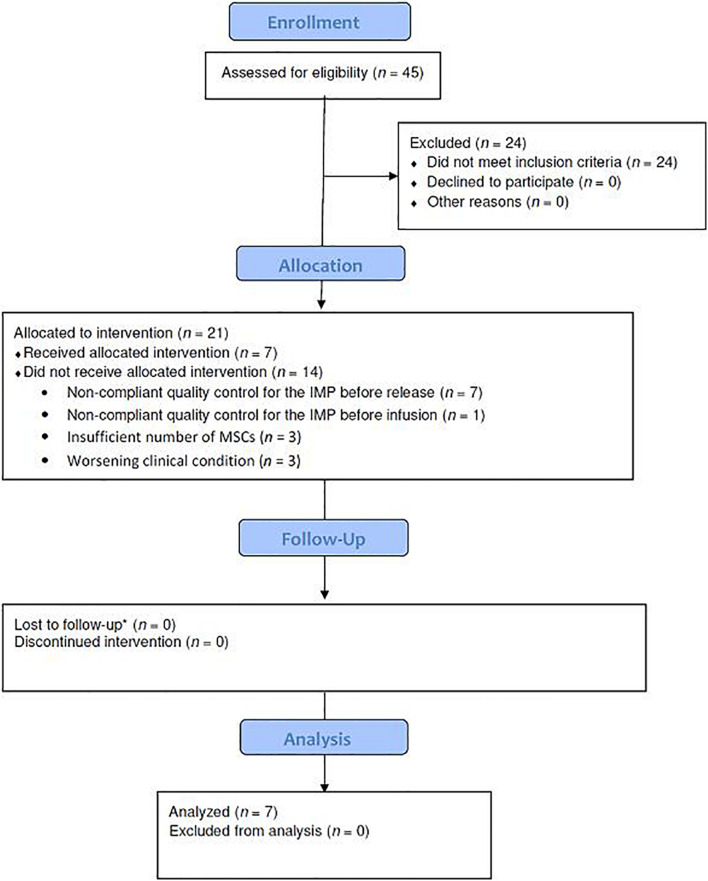
Study flow diagram showing the patient enrollment, allocation, follow-up, and analysis in the phase I study. IMP, investigational medicinal product; MSCs, mesenchymal stromal cells.

The overall results of the patient selection process are summarized in Figure 1. Of the 45 patients initially assessed for eligibility, seven patients were treated. The results of cell manufacturing in terms of MSC characteristics are shown in Table 1. The main reason for failure in obtaining a conform final product was insufficient cell growth and therefore it was unjustified to repeat the production since the same growing capacity is expected from starting material obtained from the same subject.

**TABLE 1:** Demographic, MSC characteristics, and clinical assessment data.

Demographic data	
Gender (male/female)	2/6
Age (years)	65 (60–68)
Disease duration (months)	48 (36–108)
MSC dosage and purity	
Total MSCs (×10^6^)	97.0 (77–156)
Cell dose (×10^6^)/kg	1.6 (1.0–2.0)
Purity	96.8 (95.3–98.9)
**Survival**	
Alive/total treated after 1 year from treatment	6/7
**Adverse events**	
Number of adverse events (*N* = 8)	17
Hematoma at the injection site	7
Asymptomatic spotty lesion	6
Left hemiparesis	1
Urticaria	1
Phosphenes	1
Hallucinosis	1
**MMSE**	
Baseline (*n* = 8)	25.51 (24.27–28.27)
1-month (*n* = 5)	26.49 (24.27–28.53)
12-month (*n* = 4)	24.79 (21.27–25.2)
**H&Y score ≥ 4**	
Baseline (*n* = 8)	8
1-month (*n* = 6)	6
3-month (*n* = 7)	6
6-month (*n* = 4)	4
12-month (*n* = 4)	4
**UPDRS III**	
Baseline (*n* = 8)	44.5 (31–58)
1-month (*n* = 6)	36.5 (30–48)
3-month (*n* = 7)	48 (27–55)
6-month (*n* = 4)	42.5 (39–51)
12-month (*n* = 4)	47 (40–49)
**PSP-RS**	
Baseline (*n* = 8)	46 (29–57)
1-month (*n* = 6)	40 (21–46)
3-month (*n* = 7)	44 (24–69)
6-month (*n* = 4)	49.5 (31–63)
12-month (*n* = 4)	50 (41–57)

*Data are reported as median (range). The purity of the MSCs was calculated as a percentage of CD45-/90 + /105 + cells. MSCs, mesenchymal stromal cells; MMSE, Mini-Mental State Evaluation; H&Y, Hoehn & Yahr stage; UPDRS III, Unified Parkinson’s Disease Rating Scale part III; PSP-RS, Progressive Supranuclear Palsy Rating Scale.*

### Cell Infusion

The results of the angiography performed just before cell injection are reported in Table 2. The median duration of the arterial catheterization procedure for cell infusion was 3 h and 35 min (*n* = 7; range: 3–4 h and 45 min). The preliminary angiographic study before cell infusion documented anatomic variation of the Willis circle in three of the seven patients. In one patient (Case 3), the right posterior cerebral artery originated directly from the carotid siphon. In this patient, the left carotid artery was extremely winding and therefore, MSCs were not infused into the left side of the cerebral circulation. No severe adverse events were registered during the infusion procedure, and the most common side effect was hematoma at the injection site.

**TABLE 2:** Angiographic and post-infusion BMR data (*n* = 7).

Patient	Angiography	BMR at 24 h from cell infusion	
		Number of spotty lesions	Site
Case 1	Normal	5	Insula; right superior frontal and parietal cortex, left pedunculus, left peritrigonal region
Case 2	Absence of A1 trait of the right anterior cerebral artery	3	Temporo-mesial region, optic trait, right frontal cortical–subcortical junction
Case 3	Fetal origin of the right posterior cerebral artery that directly originated from carotid siphon. Tortuosity of the left carotid artery	7	Left cerebellar hemisphere, right pons, bilateral frontal cortex, right temporal cortex, left posterior region of the lenticular nucleus, left parietal and parasagittal cortex
Case 4	Hypoplasia of A1 tract of right anterior cerebral artery	2	Pons and mesencephalus
Case 5	Normal	2	Right subcortical temporal region, right cortical parietal region
Case 6	Normal	3	Right subcortical temporal region, right cortical frontal and parietal regions
Case 7	Normal	0	None

### Safety and Efficacy Evaluation

Regarding safety, the overall results are shown in Table 1. Single patient’s data are reported in Supplementary Table 1. In addition to the previously published results (Canesi et al., 2016), the two patients treated in the second phase were living 1 year after the procedure. For one patient (Case 6), the following three adverse events were registered after cell infusion. (1) Phosphenes were observed before and during the catheterization procedure. (2) Millimeter-scale ischemic alterations in BMR signals were observed after the catheterization procedure. These lesions were not observed in the BMR 2 weeks later. (3) The patient experienced hallucinosis and reported perceiving clear images of animals or persons on a white wall, especially at twilight. This phenomenon occurred more than 48 h after cell infusion and lasted less than 12 h. No further adverse events were reported for this subject or the other patient.

The day after cell infusion, BMR showed spotty signal alterations compatible with recent ischemic lesions in one of the two patients treated in the second phase (overall six of the seven treated patients). This patient was asymptomatic. As previously reported (Canesi et al., 2016), spotty lesions were observed in the last patient of the first cohort. In addition, this patient showed ischemic alterations in the posterior segment of the left inferior peduncle of the cerebellum and in the right mesencephalon, and these symptoms were transient.

**FIGURE 2:**
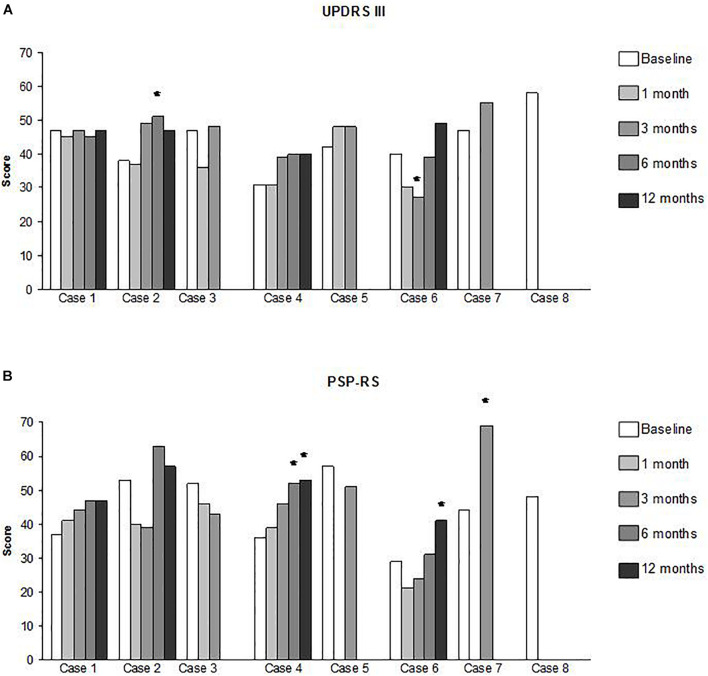
Clinical assessments data per single patient and at each time point. **(A)** UPDRS III (Unified Parkinson’s Disease Rating Scale part III, motor score); **(B)** PSP – RS (PSP Rating Scale). The star symbol (*) identifies those measures that overcome the stabilization threshold as defined in the text.

A summary of our clinical findings for efficacy is shown in Table 1 and Figure 2. Single patient’s data are also contained in Supplementary Table 1. Notably, one patient (Case 6) had an improved UPDRS III score 3 months after cell infusion (–33% of the basal value). Deterioration was recorded in another patient (Case 2) in terms of the UPDRS III score (+34% of the basal value) at the 6-month follow-up. Case 4 exhibited a deterioration in the PSP-RS score at 6 months (+44% of the basal value), which persisted at the 1-year follow-up (+47% of the basal value).

Brain magnetic resonance was performed on all patients who were going to receive cell infusion on the day before the catheterization procedure (*n* = 8). The results for all patients showed atrophy of the midbrain and dilation of the third ventricle, which is compatible with PSP diagnosis (Paviour et al., 2006). No significant change was recorded at subsequent follow-ups as measured by SPECT and PET (Supplementary Table 2).

Biomechanical evaluation at baseline was performed in all the patients who were going to receive cell infusion the week before intervention (*n* = 8). The results of the biomechanical evaluations of the last three enrolled patients are presented in Table 3. Unfortunately, only one patient (Case 6) was able to complete the 1-year follow-up. In this patient, we observed a transient clinical improvement at 3 months, which deteriorated at the subsequent follow-ups.

**TABLE 3:** Posturographic data.

Posturography measurement	Healthy controls (*N* = 6)	Case 6		Case 7	Case 8
		Baseline	6 month	Baseline	Baseline
Gender (F/M)	4/2	F	F	M	F
Age (years)	66 (61–69)	66	66	65	65
Body height (cm)	160.0 (147.0–176.1)	154.4	154.4	165.9	157.8
Foot length (cm)	24.1 (21.5–26.0)	23.3	23.3	25.1	24.1
Body weight (kg)	59.9 (55.0–70.0)	76.6*	76.6*	66.6	57.7
Base of support (cm^2^)	680.6 (503.6–736.8)	816.9*	1021.3*	958.4*	814.2*
Base width (cm)	14.2 (11.8–20.4)	28.4*	31.9*	26.3*	24.1*
Foot alignment (cm)	0.8 (0.4–1.6)	2.4*	5.0*	1.8*	1.2
Distance CoP heel (%FL)	41 (34–47)	38	45	30*	28*
Delta forces (*N*)	12.4 (4.1–50.1)	21.0	8.5	83.5*	71.7*
Ellipse area (mm^2^)	81 (24–265)	213	293*	422*	67
Axis a ellipse (mm)	6.8 (4.4–13.7)	13.1	22.8*	30.5*	9.2
Axis b ellipse (mm)	3.4 (1.7–5.7)	5.2	4.1	4.4	2.3
Eccentricity	0.89 (0.80–0.95)	0.89	0.98*	0.99*	0.96*
Mean distance (mm)	3.4 (2.0–6.5)	6.3	9.8*	13.3*	3.9
RMS distance (mm)	3.9 (2.4–7.5)	7.2	11.9*	15.7*	4.9
Median frequency (Hz)	0.5 (0.3–0.6)	0.4	0.3*	0.2*	0.5

Data are shown as the median (range) for the healthy control group and as the median of at least three assessments for the patients. *Data below/above the 10th/90th percentile of the distribution of healthy controls.

### Inflammatory Cytokine Measurements

Cytokine analysis was performed on all of the treated patients (*n* = 7; Supplementary Figure 1). The differences in cytokine serum concentrations before and after cell administration were not statistically significant (Wilcoxon matched-pairs signed-rank test). In three of the seven patients (Case 2, 3, and 4), the values of IL-8 (pro-inflammatory) were more than five times lower than those in the basal samples after cell administration (4.01, 6.66, and 25.09%, respectively).

## Discussion/Conclusion

In 2016 we already published the clinical results obtained in the first exploratory phase of our clinical protocol based on the use of MSCs in five patients affected by PSP (Canesi et al., 2016). We were therefore authorized by our competent authority to treat other three patients to complete the exploratory study. The results of the additional patients included in the exploratory phase and the reasons that prompt us not to go ahead with the following randomized phase are contained in the present manuscript. The primary finding expected from the completion of the preliminary phase of our work herein discussed is the evaluation of the risk/benefit ratio. The data gathered from the preliminary phase of our work carry important safety concerns due to the intrinsic ischemic risk of intra-arterial administration, together with encouraging efficacy aspects. The clinical assessments indeed demonstrated that all five patients in the preliminary phase I study had stable scores for at least two validated scales at their last follow-up, and one patient maintained this stabilization for 1 year. After implementing additional control measures to improve safety, in the second preliminary phase, no further severe unexpected adverse events were observed in the additional two treated patients, but numbers are too scarce for any reliable conclusion. Ischemic events in the context of this trial may be theoretically due to several factors, some of them intrinsically related to the route of administration and independent from the cells and others potentially linked to the still controversial pro-coagulant effects of MSC (Coppin et al., 2019). In the previous clinical experience with intra-arterial infusion of MSC for multiple systemic atrophy reported by Lee et al. (2012), microembolization was indeed present also in the placebo group and at a higher frequency compared to the treated group (35% vs 29%). Other factors that may be involved in microembolization/microbleeding during MSC intra-arterial administration are cell size and type (Karlupia et al., 2014), and infusion velocity (Cui et al., 2015). Also, the effect of heparin or of excipients in the final product may be involved, even though no clear evidence is reported to date. Notable, DMSO has been proven to have some toxicity on red blood cells, platelet, and vascular endothelial cells also at a concentration below 1% (Yi et al., 2017). The causal relationship between all these factors and the reported adverse events remains to be established. From the clinical point of view, it cannot remain unsaid that what we defined as asymptomatic lesions as far as we know, may have some less obvious negative impact on the clinical progression. As a conclusion, the risk/benefit balance of the approach followed in our trial has still to be improved. Regarding efficacy, unfortunately, only four of the seven treated patients were able to complete the 1-year follow-up. One patient died after an accidental fall 4 months after treatment, and two patients were not compliant to longer follow-up. In all of the four patients who completed follow-ups, we observed stabilization on at least two clinical scales until 6 months after cell infusion. In two patients, the stabilization threshold was met on only the UPDRS-III or PSP-RS. No variation was documented for PET. The overall safety and efficacy results are still inconclusive at this time because of the low number of patients and consequently the poor statistical power of the study.

At the end of the second part of the pilot phase, we decided not to proceed to the randomized phase. We made this decision primarily because the recruitment process was slow and ineffective. Consequently, we would not have been able to reach definitive conclusions regarding the risk/benefit ratio of the proposed approach in a reasonable period.

As discussed in our previous research (Giordano et al., 2014), MSCs may aid in the treatment of neurodegenerative disorders by restoring neural cell function and homeostasis and by exerting anti-apoptotic and anti-inflammatory effects on neural and glial cells, thus reducing neuroinflammation. Despite having a very promising mechanism of action, cell therapy for complex and rare diseases like PSP carries several intrinsic limitations, which affected our work. In particular, we highlight the difficulty in identifying the best candidates for MSC treatment among patients that suffer from heterogeneous and rapidly progressive disorders, such as PSP.

In spite of these challenges, the experience gained in our trial is valuable for the design, planning, and management of future clinical trials. Indeed, finding novel therapeutic approaches for orphan diseases remains a priority and cell-based regenerative therapies represent a promising tool in this field (Tambuyzer et al., 2020). Among other cell types, MSCs have dramatic anti-inflammatory and immunomodulatory properties (Marigo and Dazzi, 2011). Moreover, an inflammatory environment is an important determinant of the efficacy of cellular therapy (van Megen et al., 2019). Interestingly, in our study, two of the three patients (Cases 2 and 3) who showed significant reductions in serum IL-8 concentrations also had improvements in both UPDRS and PSP-RS at the 1-month follow-up after treatment, and they showed improvements in specific symptoms (neck pain, ocular motility, and photophobia). These results were previously published (Canesi et al., 2016). Due to the insufficient sample size, we cannot consider these results as more than anecdotal events, but the short-term effect of cell-based therapies on specific symptoms should be addressed in further studies. In addition, it will be important to examine the effect of repeated cell administration.

Another important issue for the development of phase II efficacy studies is applying the right diagnostic classifications for suitable patients. The Movement Disorder Society (MDS) has recently identified diagnostic criteria for PSP (Höglinger et al., 2017) and defined eight predominance types: PSP-RS, Richardson syndrome; PSP-PI, predominant postural instability; PSP-OM, predominant ocular motor dysfunction; PSP-P, predominant parkinsonism; PSP-PGF, progressive gait freezing; PSP-CBS, predominant corticobasal syndrome; PSP-F, predominant frontal presentation, and PSP-SL, predominant speech/language disorder. Notably, all of the patients enrolled in our study were classified as having Richardson’s syndrome. The correct classification of the patients in a context of a clinical trial is not a trivial problem and it can affect the reliability of efficacy evaluation. That’s why, to overcome the problem of multiple diagnostic allocations of a single patient, guidelines are now available for the application of the new criteria in PSP patients who show symptoms of more than one functional domain (so-called MAX rules for multiple allocation extinction; Grimm et al., 2019). The MDS criteria for PSP also introduced the category “probable 4-repeat (4R)-tauopathy” for joint clinical diagnosis of PSP and corticobasal degeneration. These new diagnostic categories and classifications have high specificity and may be suitable for the recruitment of patients with PSP and corticobasal degeneration into therapeutic trials.

The scarcity of validated *in vivo* disease-specific biomarkers for both the diagnosis and the assessment of disease progression is another important bottleneck for early phase interventional trials in PSP. To improve the reliability of our results, we used multiple clinical scales and empirically considered longitudinal changes of ±30% as significant in terms of effectiveness. This cut-off value was defined by comparisons with cohorts of patients with the same characteristics (Litvan and Kong, 2014). We also performed a detailed biomechanical evaluation and state-of-the-art imaging studies with the aim of documenting the rate of change after the experimental treatment. For future efficacy trials, *in vivo* imaging biomarkers (e.g., Tau-PET) as well as multiple CSF and blood biomarkers should be considered.

Our study revealed critical considerations in cell manufacturing when using autologous cells from aged and diseased subjects. In our experience, 8 out of the 12 treatment failures (67%) were due to cell-related causes (low numbers or non-compliant quality controls). We have previously demonstrated that several disease-related factors affect the cell growth potential of MSCs from PSP patients (e.g., microtubule instability may lead to altered paracrine function and mitochondrial dysfunction may lead to altered cell growth; Angelova et al., 2018; Calogero et al., 2018). These factors may explain the deviations we recorded in cell production.

In performing this phase I study, we identified several critical issues in the production and use of ATMPs for rare diseases in autologous settings. This work encourages further studies using innovative designs (e.g., adaptive studies), employing allogenic cells as starting material and a-cellular therapy approaches. In addition, our experience supports the use of technological innovations for safer cell delivery.

In conclusion, the main question addressed in our work is “How can we safely treat neurodegeneration with cell therapy”? Indeed, the anti-inflammatory properties of MSCs are at the moment exploited in many pathological contexts, but they are still poorly applied in neurodegenerative disorders. In particular, at the date of submission of the present article, there is only one study registered at clinicaltrials.gov using BM-derived MSCs for the treatment of several neurologic diseases including PSP (ClinicalTrials.gov Identifier: NCT02795052) and on June 1, 2021, a search in Pubmed using the key-words “Progressive Supranuclear Palsy,” “Parkinsonisms,” “Cell,” and “Clinical trial” gives rise to 10 articles and only two of them are pertinent, both from our study group. Therefore, it is clear that the many bottlenecks and concerns that we have herein underlined discourage researchers to study the clinical effects of cell therapy in neurodegenerative disorders. Our pioneeristic experience may be of great help for people working in the search of advanced therapeutic tools for still unmet medical needs.

## Data Availability Statement

The raw data supporting the conclusions of this article will be made available by the authors, without undue reservation.

## Ethics Statement

The studies involving human participants were reviewed and approved by the Ethics Committee of Fondazione IRCCS Ca’ Granda Ospedale Maggiore Policlinico (Italy). The patients/participants provided their written informed consent to participate in this study. Written informed consent was obtained from the individual(s) for the publication of any potentially identifiable images or data included in this article.

## Author Contributions

RG and MC made substantial contributions to the conception, organization, and execution of this work; to the writing of the manuscript and to its review and critique. Specifically, RG established good manufacturing practices (GMP) procedures for MSCs including validation, production, and quality controls; wrote protocols; submitted the trial to regulatory authorities; and coordinated the project. MC performed patient selection, clinical evaluations, and follow-up evaluations. MI performed cell administration. II and CP performed multifactorial movement analysis and analyzed the results. II aided in revising the manuscript. RB and GiorM defined and performed the PET/SPECT procedures and analyzed the results. GiovM and RC performed clinical assessments of the patients before, during, and after treatment and described and reported all adverse events. AR and VC performed neuropsychological assessments. LL, TM, and MV aided in establishing GMP for MSC, including validation, production, and quality controls. SB helped prepare and submit the trial to regulatory authorities and collected patient data during the study follow-up. EM and CL contributed to MSC production and quality control. DV performed bone marrow aspiration and hematological patient assessments. AR-O performed cytokine studies, interpreted data, and helped revise the manuscript. GP made substantial contributions to the conception, design, organization, and execution of this research project. Specifically, GP conceived the clinical trial, contributed to the study design, and aided in writing the research protocol. All authors contributed to the article and approved the submitted version.

## Conflict of Interest

The authors declare that the research was conducted in the absence of any commercial or financial relationships that could be construed as a potential conflict of interest.

## Publisher’s Note

All claims expressed in this article are solely those of the authors and do not necessarily represent those of their affiliated organizations, or those of the publisher, the editors and the reviewers. Any product that may be evaluated in this article, or claim that may be made by its manufacturer, is not guaranteed or endorsed by the publisher.

## Footnotes

1
www.graphpad.com

## Supplementary Material

Supplementary Figure 1 Serum cytokine levels in PSP patients before and after MSC administration. Dot plot diagrams comparing serum concentrations for each cytokine (Wilcoxon matched-pairs signed-rank test). **(a)** IL-6 (*p* = 0.375), **(b)** IL-8 (*p* = 0.5781), **(c)** TNF-α (*p* = 0.2969), **(d)** IL-1β (*p* = 0.375), **(e)** IL-5 (*p* = 0.125), **(f)** IL-10 (*p* = 0.0938), and **(g)** IFNγ (*p* = 0.0781). In one patient, the pre-treatment IL-5 serum concentration was below the quantification limit.

Supplementary Table 1 Individual patient data at each time point: demographical data, treatment details (administered cell dose and purity), baseline and follow-up neuropsychological assessments by Mini-Mental State Evaluation (MMSE) and clinical scoring, and serum cytokine level. n.a. = not available.

Supplementary Table 2 SPECT data (*n* = 3). Specific striatal dopamine uptake transporter binding of [I-123] ioflupane. Binding was calculated for the left and right striatum, putamen, and caudate nucleus using the following formula, where ROI is the region of interest: [(mean counts in specific ROI) − (mean counts in occipital ROI)]/(mean counts in occipital ROI). n.a.: not available; L: left; R: right.
