# Supplementary figures and images for: Safety and Effectiveness of Cell Therapy in Neurodegenerative Diseases: Take-Home Messages From a Pilot Feasibility Phase I Study of Progressive Supranuclear Palsy
Source: Front Neurosci. 2021 Oct 12;15:723227. doi: 10.3389/fnins.2021.723227 (PMC8546103; doi:10.3389/fnins.2021.723227)

a)

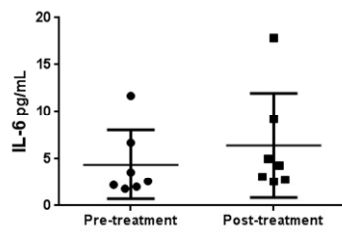

b)

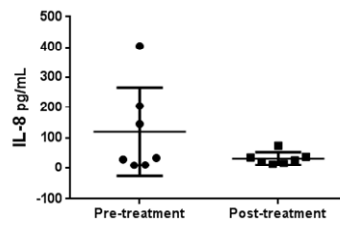

c)

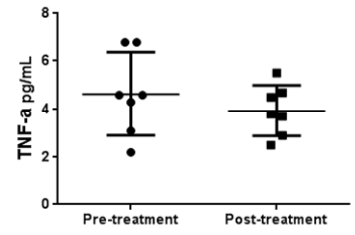

d)

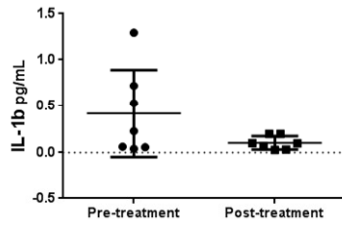

e)

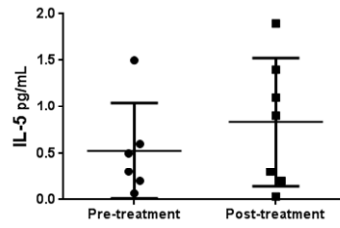

f)

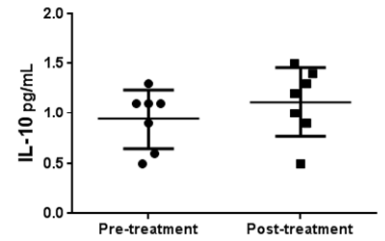

g)

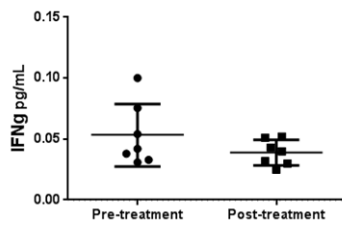

Supplement: Supplementary Figure 1 — Serum cytokine levels in PSP patients before and after MSC administration. Dot plot diagrams comparing serum concentrations for each cytokine (Wilcoxon matched-pairs signed-rank test). (a) IL-6 (p = 0.375), (b) IL-8 (p = 0.5781), (c) TNF-α (p = 0.2969), (d) IL-1β (p = 0.375), (e) IL-5 (p = 0.125), (f) IL-10 (p = 0.0938), and (g) IFNγ (p = 0.0781). In one patient, the pre-treatment IL-5 serum concentration was below the quantification limit. [file Image_1.pdf]
