# Supplementary material for: Safety and Effectiveness of Cell Therapy in Neurodegenerative Diseases: Take-Home Messages From a Pilot Feasibility Phase I Study of Progressive Supranuclear Palsy
Source: Front Neurosci. 2021 Oct 12;15:723227. doi: 10.3389/fnins.2021.723227 (PMC8546103; doi:10.3389/fnins.2021.723227)
Supplement: Supplementary Table 1 — Individual patient data at each time point: demographical data, treatment details (administered cell dose and purity), baseline and follow-up neuropsychological assessments by Mini-Mental State Evaluation (MMSE) and clinical scoring, and serum cytokine level. n.a. = not available. [file Table_1.doc]

***Supplementary Table 1 Single patients’ description****.*Demographical data, treatment details, baseline and follow-up neuropsychological assessments by mini-mental state evaluation (MMSE) and clinical scoring using three different scales: Hoehn-Yahr stage (H&Y), Unified Parkinson’s Disease Rating Scale part III (UPDRS III); PSP rating score (PSP-RS). For UPDRS and PSP-RS the values are reports as absolute value and percentage of variation from baseline (in brackets). Stabilization, as defined in the text, was registered at the last follow-up in all the patients. N.a.: not available.

|  | **Case 1** | **Case 2** | **Case 3** | **Case 4** | **Case 5** | **Case 6** | **Case 7** | **Case 8** |
| --- | --- | --- | --- | --- | --- | --- | --- | --- |
| **Demographic data** |  |  |  |  |  |  |  |  |
| Gender | M | F | F | F | F | F | M | F |
| Age (years) | 60 | 66 | 65 | 65 | 68 | 65 | 65 | 64 |
| Disease duration (years) | 8 | 3 | 4 | 4 | 4 | 4 | 9 | 7 |
| **Treatment** |  |  |  |  |  |  |  |  |
| Total cell number (x10^6^) | 110 | 82 | 156 | 125 | 97 | 77 | 112.5 | - |
| Cell dose (x10^6^/kg) | 1.4 | 1.7 | 2 | 1.8 | 1.2 | 1.0 | 1.5 | - |
| Purity (%) | 98.9 | 97.9 | 96.4 | 96.4 | 95.3 | 98.8 | 97.1 | - |
| **MMSE** |  |  |  |  |  |  |  |  |
| Baseline | 27.49 | 28.27 | 25.49 | 24.27 | 25.53 | 27 | 25 | 25 |
| 1-month | 27.49 | 25.27 | 26.49 | 24.27 | 28.53 | 27 | n.a. | n.a. |
| 12-month | 26.49 | 25.03 | n.a. | 21.27 | n.a. | 25 | n.a. | n.a. |
| **H&Y** |  |  |  |  |  |  |  |  |
| Baseline | 4/5 | 4/5 | 4/5 | 4/5 | 4/5 | 4/5 | 4/5 | 4/5 |
| 1-month | 4/5 | 4/5 | 4/5 | 4/5 | 4/5 | 4/5 | n.a. | n.a. |
| 3-month | 4/5 | 4/5 | 4/5 | 4/5 | 4/5 | 3/5 | 4/5 | n.a. |
| 6-month | 4/5 | 4/5 | n.a. | 4/5 | n.a. | 5/5 | n.a. | n.a. |
| 12-month | 4/5 | 4/5 | n.a. | 4/5 | n.a. | 4/5 | n.a. | n.a. |
| **UPDRS III** |  |  |  |  |  |  |  |  |
| Baseline | 47 | 38 | 47 | 31 | 42 | 40 | 47 | 58 |
| 1-month | 45 (-4%) | 37 (-3%) | 36 (-23%) | 31 (0%) | 48 (+14%) | 30 (-25%) | n.a. | n.a. |
| 3-month | 47 (0%) | 49 (+29%) | 48 (+2%) | 39 (+26%) | 48 (+14%) | 27 (-33%) | 55 (+17%) | n.a. |
| 6-month | 45 (-4%) | 51 (+34%) | n.a. | 40 (+29%) | n.a. | 39 (-3%) | n.a. | n.a. |
| 12-month | 47 (0%) | 47 (+24%) | n.a. | 40 (+29%) | n.a. | 49 (+23%) | n.a. | n.a. |
| **PSP-RS** |  |  |  |  |  |  |  |  |
| Baseline | 37 | 53 | 52 | 36 | 57 | 29 | 44 | 48 |
| 1-month | 41 (+11%) | 40 (-25%) | 46 (-12%) | 39 (+8%) | n.a. | 21 (-28%) | n.a. | n.a |
| 3-month | 44 (+19%) | 39 (-26%) | 43 (-17%) | 46 (+28%) | 51 (-11%) | 24 (-17%) | 69 (+57%) | n.a. |
| 6-month | 47 (+27%) | 63 (+19%) | n.a. | 52 (+44%) | n.a. | 31 (+7%) | n.a. | n.a. |
| 12-month | 47 (+27%) | 57 (+8%) | n.a. | 53 (+47%) | n.a. | 41 (+41%) | n.a. | n.a. |
| **Serum cytokines (pg/mL)** |  |  |  |  |  |  |  |  |
| IFN-gamma |  |  |  |  |  |  |  |  |
| Basal | 0.076 | 0.031 | 0.042 | 0.054 | 0.038 | 0.033 | 0.1 | - |
| 72 hours after | 0.052 | 0.040 | 0.032 | 0.051 | 0.025 | 0.030 | 0.043 | - |
| IL-10 |  |  |  |  |  |  |  |  |
| Basal | 1.1 | 0.9 | 1.1 | 0.6 | 1.3 | 1.1 | 0.5 | - |
| 72 hours after | 1.0 | 1.4 | 1.3 | 0.9 | 1.5 | 1.2 | 0.5 | - |
| IL-1 beta |  |  |  |  |  |  |  |  |
| Basal | 1.300 | 0.058 | 0.229 | 0.719 | 0.063 | 0.041 | 0.530 | - |
| 72 hours after | 0.034 | 0.200 | 0.098 | 0.069 | 0.100 | 0.200 | 0.029 | - |
| IL-5 |  |  |  |  |  |  |  |  |
| Basal | 0.6 | 1.5 | 0.5 | 0.2 | 0.067 | 0.3 | undetectable | - |
| 72 hours after | 1.1 | 1.4 | 0.9 | 1.9 | 0.200 | 0.3 | 0.033 | - |
| IL-6 |  |  |  |  |  |  |  |  |
| Basal | 3.6 | 1.8 | 11.7 | 2.2 | 6.7 | 2.6 | 2.0 | - |
| 72 hours after | 2.8 | 5.0 | 9.2 | 4.3 | 17.9 | 3.1 | 2.6 | - |
| IL-8 |  |  |  |  |  |  |  |  |
| Basal | 204.9 | 10.1 | 145.3 | 404.5 | 35.2 | 30.3 | 11.1 | - |
| 72 hours after | 13.6 | 27.9 | 36.5 | 16.2 | 39.0 | 74.8 | 20.8 | - |
| TNF-alfa |  |  |  |  |  |  |  |  |
| Basal | 6.8 | 4.6 | 6.8 | 2.2 | 4.6 | 4.3 | 3.1 | - |
| 72 hours after | 3.7 | 3.8 | 5.5 | 2.5 | 4.5 | 4.7 | 2.9 | - |
