# Supplementary material for: Safety and Effectiveness of Cell Therapy in Neurodegenerative Diseases: Take-Home Messages From a Pilot Feasibility Phase I Study of Progressive Supranuclear Palsy
Source: Front Neurosci. 2021 Oct 12;15:723227. doi: 10.3389/fnins.2021.723227 (PMC8546103; doi:10.3389/fnins.2021.723227)
Supplement: Supplementary Table 2 — SPECT data (n = 3). Specific striatal dopamine uptake transporter binding of [I-123] ioflupane. Binding was calculated for the left and right striatum, putamen, and caudate nucleus using the following formula, where ROI is the region of interest: [(mean counts in specific ROI) − (mean counts in occipital ROI)]/(mean counts in occipital ROI). n.a.: not available; L: left; R: right. [file Table_2.doc]

**Supplementary Table 2. SPECT data (*n* = 3).**

| **Brain Region** | **Case 6** | | **Case 7** | **Case 8** | | |
| --- | --- | --- | --- | --- | --- | --- |
|  | **Baseline** | **12-month** | **Baseline** | | **Baseline** |  |
| R striatum | 1.25 | 1.15 | 0.33 | | 0.33 |  |
| L striatum | 1.16 | 1.09 | 0.37 | | 0.22 |  |
| R caudate nucleus | 1.64 | 1.35 | 0.38 | | 0.31 |  |
| L caudate nucleus | 1.40 | 1.32 | 0.51 | | 0.31 |  |
| R putamen | 0.87 | 0.92 | 0.30 | | 0.29 |  |
| L putamen | 0.94 | 0.89 | 0.24 | | 0.13 |  |
